# Biocalorimetry-aided monitoring of fungal pretreatment of lignocellulosic agricultural residues

**DOI:** 10.1007/s00253-024-13234-y

**Published:** 2024-06-25

**Authors:** Hieu Linh Duong, Sven Paufler, Hauke Harms, Thomas Maskow, Dietmar Schlosser

**Affiliations:** 1 Department of Applied Microbial Ecology, Helmholtz-Centre for Environmental Research-UFZ, Permoserstraβe 15, 04318 Leipzig, Germany; 2 Vietnamese-German University (VGU), Ring Road 4, Quarter 4, Thoi Hoa Ward, Ben Cat City, Binh Duong Province Vietnam

**Keywords:** Biocalorimetry, Fungi, Metabolic heat, Lignocellulose, Pretreatment, Wheat straw

## Abstract

**Abstract:**

The present study aimed to investigate whether and how non-invasive biocalorimetric measurements could serve for process monitoring of fungal pretreatment during solid-state fermentation (SSF) of lignocellulosic agricultural residues such as wheat straw. Seven filamentous fungi representing different lignocellulose decay types were employed. Water-soluble sugars being immediately available after fungal pretreatment and those becoming water-extractable after enzymatic digestion of pretreated wheat straw with hydrolysing (hemi)cellulases were considered to constitute the total bioaccessible sugar fraction. The latter was used to indicate the success of pretreatments and linked to corresponding species-specific metabolic heat yield coefficients (*Y*_*Q*/*X*_) derived from metabolic heat flux measurements during fungal wheat straw colonisation. An *Y*_*Q*/*X*_ range of about 120 to 140 kJ/g was seemingly optimal for pretreatment upon consideration of all investigated fungi and application of a non-linear Gaussian fitting model. Upon exclusion from analysis of the brown-rot basidiomycete *Gloeophyllum trabeum*, which differs from all other here investigated fungi in employing extracellular Fenton chemistry for lignocellulose decomposition, a linear relationship where amounts of total bioaccessible sugars were suggested to increase with increasing *Y*_*Q*/*X*_ values was obtained. It remains to be elucidated whether an *Y*_*Q*/*X*_ range being optimal for fungal pretreatment could firmly be established, or if the sugar accessibility for post-treatment generally increases with increasing *Y*_*Q*/*X*_ values as long as “conventional” enzymatic, i.e. (hemi)cellulase-based, lignocellulose decomposition mechanisms are operative. In any case, metabolic heat measurement–derived parameters such as *Y*_*Q*/*X*_ values may become very valuable tools supporting the assessment of the suitability of different fungal species for pretreatment of lignocellulosic substrates.

**Key points:**

*• Biocalorimetry was used to monitor wheat straw pretreatment with seven filamentous fungi.*

*• Metabolic heat yield coefficients (Y*_*Q/X*_ *) seem to indicate pretreatment success.*

*• Y*_*Q/X*_ *values may support the selection of suitable fungal strains for pretreatment.*

**Supplementary Information:**

The online version contains supplementary material available at 10.1007/s00253-024-13234-y.

## Introduction

Pretreatment of lignocellulosic biomass is a prerequisite for the efficient utilisation of its polysaccharide fraction, yielding fermentable sugars as a basis for the subsequent production of bioenergy and various useful chemicals (Wan and Li 2012; Singh et al. 2014). However, various non-biological pretreatment methods also produce inhibitory by-products (Jönsson and Martín 2016) and place high demands for energy and/or chemicals (Mosier et al. 2005; Baruah et al. 2018). In this regard, biological pretreatment is an environmentally friendly technique, which concomitantly combines advantages such as mild reaction conditions, the specificity of enzymatic hydrolysis, the avoidance of inhibitory by-products, and low energy requirements (Singh et al. 2014; Sindhu et al. 2016; Bhatia et al. 2017; Baruah et al. 2018).

Fungal solid-state fermentation (SSF) is particularly promising for the efficient biological pretreatment of lignocellulosic agricultural wastes and the qualification of these materials for diverse biorefinery applications. White-rot basidiomycetes can degrade all plant cell wall components and specifically mineralise lignin to CO_2_ and H_2_O with the help of lignin-modifying peroxidases and diverse enzymes acting on crystalline cellulose (Riley et al. 2014). Pretreatment with white-rot fungi was previously reported to provide several advantages. For example, wheat straw pretreatment using the white-rot basidiomycetes *Irpex lacteus* and strain Euc-1 can increase the accessibility of cellulose towards enzymatic hydrolysis by about three and four times, respectively (Dias et al. 2010). More than 20% increase in the sugar yield was observed for red pine chips pretreated with *Stereum hirsutum* prior to enzymatic hydrolysis, as compared to the untreated lignocellulosic material (Lee et al. 2007). Pretreatment of sugarcane bagasse with the white-rot fungus *Ceriporiopsis subvermispora* considerably increased the cellulose digestibility, finally yielding about 47% of the glucose that potentially could be derived upon hydrolysis of the applied substrate (Machado and Ferraz 2017).

Brown-rot basidiomycetes act on lignocellulose with the help of non-enzymatic low-molecular-mass metabolites in combination with Fe and modify lignin to a polymeric brown residue (Goodell 2003; Riley et al. 2014), while rapidly depolymerising cellulose in the early stages of wood decay (Chen et al. 2010). The brown-rot fungus *Gloeophyllum trabeum* was evaluated with respect to its potential for the biological pretreatment of wheat straw. Compared to the untreated substrate, fungal pretreatment resulted in an increase in the glucose yield by about 26%, with no quantifiable lignin removal being observed (Hermosilla et al. 2018).

Soft rot fungi can extensively degrade cellulose and hemicellulose and slightly modify lignin during plant cell wall decay (Chen et al. 2010). Karpe et al. (2015) employed the soft-rot ascomycete *Penicillium chrysogenum* for SSF of winery-derived biomass waste and reported fungal metabolism of the majority of plant biomass sugars, including conversion of pentoses to alcohols. Also, fungal degradation of tannins and lignins was observed. Such properties render soft-rot fungi potentially interesting for biorefinery purposes.

Several authors have evaluated and compared the effectiveness of lignocellulose pretreatment, using different types of decay fungi. For example, Shrestha et al. (2009) used a white-rot (*Phanerochaete chrysosporium*), a brown-rot (*G. trabeum*), and a soft-rot fungus (*Trichoderma reesei*) to pretreat corn fibre, followed by simultaneous saccharification and yeast fermentation of the corresponding hydrolysates to ethanol. Approximately 8, 9, and 5 g of ethanol per 100 g corn fibre could be achieved upon pretreatment using the white-rot, brown-rot, and soft-rot fungus, respectively, with the highest ethanol yield obtained representing 35% of the yield that could theoretically be achieved from starch and cellulose in corn fibre. Singh et al. (2014) observed efficient wheat straw pretreatment with three ascomycetes of the genera *Acephala* and *Stachybotrys* (constructed wetland isolates) and two white-rot basidiomycetes (*Hypholoma fasciculare* and *Stropharia rugosoannulata*), where total amounts of water-extractable sugars increased by more than 50% and sometimes up to 150% above the value obtained from the untreated control.

Calorimetry is a smart tool to track fungal activity as it provides real-time metabolic information, does not affect growth and product formation processes, and can serve for the delivery of thermodynamic state variables for fungal activity prediction (Duong et al. 2022b). Previous studies have already established biocalorimetry for process monitoring in order to identify unexpected metabolic events (Maskow and Kleinsteuber 2004), to record metabolic shifts (Duboc et al. 1998; Maskow and Babel 1998), to control the conversion of toxic substrates into valuable products such as biopolyesters, or to protect compounds in fed-batch or a continuous manner (Maskow and Babel 2001; Maskow et al. 2006; Rohde et al. 2016). In previous studies we have applied the non-invasive measurement of metabolic heat fluxes to monitor fungal activity during the colonisation of wheat straw, which was used as a solid lignocellulosic agricultural residue of global relevance (Duong et al. 2022a,b). Fungal biomass yields observed during fungal growth on wheat straw were strongly correlated with the released metabolic heat, which enabled to determine a range of species-specific growth-related activity parameters being indicative of different fungal strategies employed during resource utilisation (Duong et al. 2022b). For example, one of these parameters (the metabolic heat yield coefficient *Y*_*Q*/*X*_, i.e. the metabolic heat released per increase of fungal biomass unit) can be considered an indicator for the degree of resource investment into fungal biomass vs. other functional attributes such as extracellular enzymes contributing to lignocellulose decomposition (Duong et al. 2022b).

Based on the aforementioned findings, the present study aimed to investigate whether and how non-invasive biocalorimetric measurements could serve for process monitoring of fungal pretreatment during SSF of lignocellulosic agricultural residues such as wheat straw. For this, a range of fungi representing different types of lignocellulose decay was employed for wheat straw pretreatment using SSF. Water-extractable sugar fractions (assumed to represent mono-, di-, oligo-, or polysaccharides or combinations thereof) in differently treated lignocellulose fractions were analysed, used to characterise the efficiency of the pretreatment/SSF processes with respect to sugar accessibility for subsequent use. These fractions were linked to corresponding species-specific *Y*_*Q*/*X*_ values. *Y*_*Q*/*X*_ is a measure of the metabolic heat released (in J) to form a certain amount of biomass (in g) and was calorimetrically quantified as described before (Duong et al. 2022a,b). In order to cover a broad range of potential SSF applications, fungi dwelling in diverse habitats and employing different strategies in utilising lignocellulosic substrates were used. The wine-cap mushroom *Stropharia rugosoannulata* served as a litter decaying and comparatively slowly growing white-rot reference strain (Singh et al. 2014; Duong et al. 2022a). The basidiomycete *G. trabeum* was chosen as a brown-rot representative (Goodell 2003; Krueger et al. 2015; Duong et al. 2022b). *Schizophyllum commune* was selected as an intermediate between brown-rot and white-rot fungi, lacking lignin-degrading peroxidases (like brown-rot fungi) but possessing enzymes acting on crystalline cellulose (like white-rot fungi) (Riley et al. 2014; Duong et al. 2022b). The soft-rot ascomycetes *T. reesei*, *Stachybotrys chlorohalonata*, and *P. chrysogenum* and the mucoromycete *Gongronella butleri* were also included (Duong et al. 2022a,b).

## Materials and methods

### Chemicals and other materials

All chemicals were of analytical grade (gradient grade in the case of chromatography solvents), unless stated otherwise. 2,2′-Azinobis-(3-ethylbenzothiazoline-6-sulfonic acid) (ABTS, purity > 98%) was obtained from AppliChem (Darmstadt, Germany). All other chemicals were purchased from Merck, Sigma-Aldrich and Th. Geyer GmbH (Renningen, Germany). The enzyme mixtures Celluclast 1.5 L and Viscozyme L were obtained from Sigma-Aldrich (Merck Group, Darmstadt, Germany). Celluclast 1.5 L, a cellulase from *T. reesei*, had an enzymatic activity of 756 glucanase units (GU)/g and was delivered at a concentration of 1.22 g/mL. Viscozyme L, a commercial cellulolytic enzyme mixture from *Aspergillus* sp., is a blend of *beta*-glucanases, pectinases, hemicellulases, and xylanases. According to the manufacturer (Novozymes Corp., Bagsvӕrd, Denmark) the main enzymatic activity of Viscozyme L was represented by *beta*-glucanase at 108 GU/g, corresponding to a concentration of 1.21 g/mL.

### Source and maintenance of fungal strains

The investigated fungi included the species *S. chlorohalonata*, *S. rugosoannulata* (Duong et al. 2022a), *S. commune*, *G. trabeum*, *T. reesei*, *P. chrysogenum*, and *G. butleri* (Duong et al. 2022b). *S. chlorohalonata* A-2008–2 (DSM 27588), *S. rugosoannulata* (DSM 11372), *G. trabeum* (DSM 1398), and *P. chrysogenum* (DSM 848) were obtained from the strain collection of the Department of Applied Microbial Ecology at the Helmholtz Centre for Environmental Research-UFZ (Leipzig, Germany). They are also available from the German Collection of Microorganisms and Cell Cultures (DSMZ; Braunschweig, Germany). *G. butleri* (DSM 2917), *S. commune* (DSM 11223), and *T. reesei* (DSM 769) were derived from the DSMZ. The fungal strains were maintained on 2% (w/v) malt extract agar plates (1.5% agar; pH 5.7) at 28 °C in the dark.

### Fungal cultivations on wheat straw and sample preparation for non-calorimetric analytical procedures

**Fig. 1:**
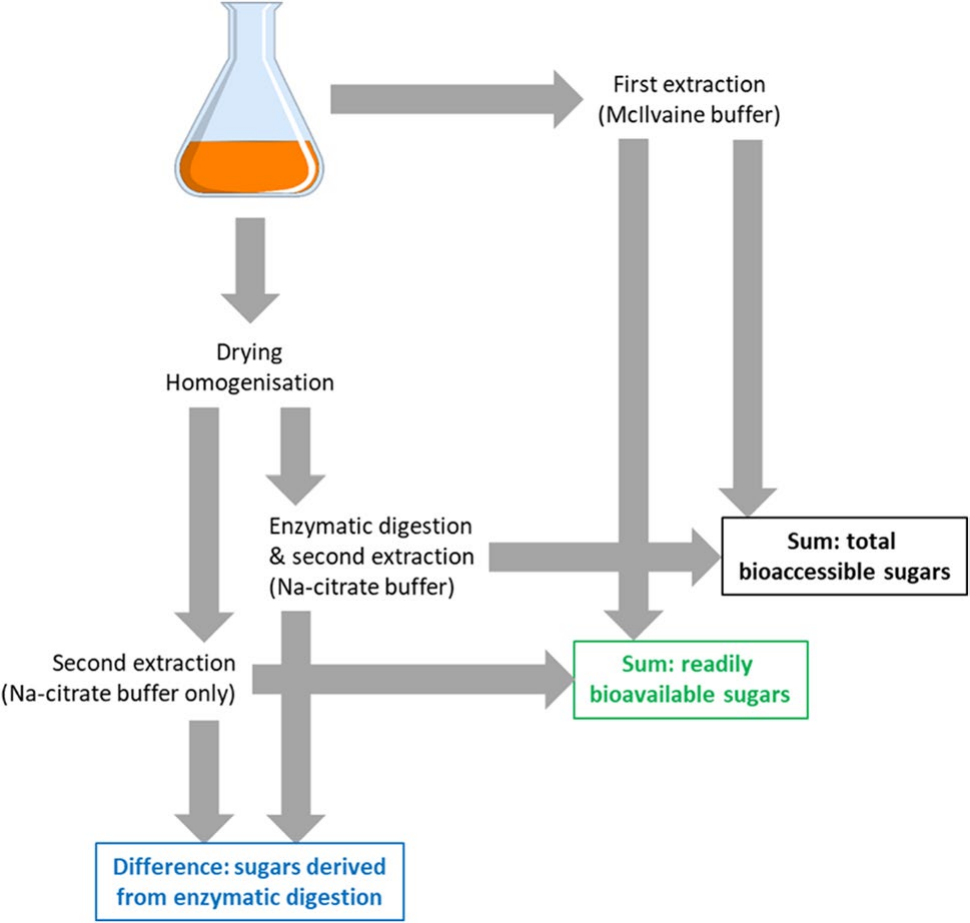
Schematic overview of aqueous extractions and enzymatic digestion of lignocellulosic samples and the sugar fractions derived thereof

Wheat straw pretreatment through axenic fungal cultivation and the subsequent extraction of water-soluble compounds are comprehensively described in Duong et al. (2022a,b). Briefly, fungi were grown on milled and prewetted sterile wheat straw (0.5 g dry mass, about 2 mm particle size) in previously sterilised calorimetric polypropylene vials (total volume 40 mL) equipped with venting PTFE membrane screw caps. For preparation of fungal inocula, agar plugs (derived from the edges of fungal colonies grown on malt extract agar plates as described above) were homogenised in 2% malt extract medium (one agar plug per 1 mL of malt extract medium) with the help of an ULTRA-TURRAX® (IKA-Werke GmbH & Co. KG, Staufen, Germany). Thereafter, 0.5 mL of the resulting fungal suspension was used to inoculate one calorimetric vial, respectively. After inoculation, vials were closed with the sterile venting membrane screw caps mentioned before. Further vials containing untreated (i.e. without autoclaving and fungal inoculation) and autoclaved wheat straw (without fungal inoculation), respectively, were also established for comparison. For fungal cultivation and recording of calorimetric data, the vials were placed in independent measurement channels in a MC CAL isothermal microcalorimeter (C3 Prozess- und Analysentechnik GmbH, Haar b. München, Germany). The working temperature was set to 28.00 ± 0.01 °C. All further details can be retrieved from Duong et al. (2022a,b). After total incubation periods of either 32 (in the case of S*. rugosoannulata* and *S. chlorohalonata*) or 20 days (all other fungi), the vials were harvested and stored at − 20 °C. Thereafter, 0.1 M McIlvaine buffer (McIlvaine 1921) (pH 7.0) was applied in order to extract water-soluble compounds from the solid materials in a step in the following referred to as “first extraction” (please also refer to Fig. 1). Aqueous supernatants resulting from the extractions were stored at − 20 °C until further analyses described below. Total dry masses of solid fractions remaining from the first extraction were determined as previously described (Singh et al. 2014; Duong et al. 2022a).

### Enzymatic digestion of samples

Solids remaining from total dry mass determination were homogenised with a ball mill (Pulverisiette 23; Fritsch, Idar-Oberstein, Germany) at 50 oscillations per second for 5 min and stored at ambient temperature under dry conditions in the dark until enzymatic digestion (hydrolysis) was carried out. The commercial enzyme preparations Celluclast 1.5 L (a cellulase) and Viscozyme L (a blend of beta-glucanases, pectinases, hemicellulases and xylanases) were applied based on previously published data (López-Gutiérrez et al. 2021) in mixture at 121 and 15.8 GU/g dry lignocellulosic solid, respectively. Enzymatic hydrolysis was carried out in 0.1 M Na-citrate buffer (pH 4.8) containing 0.2 g/L tetracycline in addition in order to suppress bacterial growth in the reaction mixtures. Lignocellulosic solid samples were applied at 2.5% (w/v). Incubations were carried out at 150 rpm and 40 °C for 18 h, hereby concomitantly extracting water-soluble sugar fractions (further on referred to as “second extraction”; Fig. 1). After that, samples were immediately frozen at − 20 °C and stored at this temperature until analysis. Additional lignocellulosic solids were always incubated in parallel, employing Na-citrate buffer in the absence of hydrolysing enzymes. Samples of untreated (not autoclaved) and autoclaved wheat straw without fungal pretreatment were also incubated in the presence and absence of hydrolysing enzymes, respectively, and served as controls. All incubations described above were carried out in triplicate.

### Overview of the sugar fractions obtained from the different aqueous extraction steps and enzymatic digestion

After thawing the samples obtained from different aqueous extraction steps and enzymatic digestion as described above, these were centrifuged at 4 °C and 20,817 × g for 10 min (Eppendorf centrifuge 5430-R, rotor type FA-45–30-11; Eppendorf, Hamburg, Germany). The resulting aqueous supernatants were kept at − 20 °C for further analyses, which included the determination of total reducing sugars based on the dinitrosalicylic acid (DNSA) method, the determination of total sugars after acidic hydrolysis using the phenol–sulphuric acid method, and the determination of glucose, xylose, and fructose with the help of ultra-performance liquid chromatography (UPLC) as described below, respectively. A schematic summary of the different extraction and enzymatic digestion steps and the sugar fractions derived thereof is illustrated in Fig. 1. Three important sugar fractions were defined. The “readily bioavailable sugar” fraction refers to the sum of sugars derived from the first and the second extraction without enzymatic digestion. In a technical process such as biogas or bioethanol production from lignocellulosic substrates, this water-soluble sugar fraction could be expected to be immediately available to fermenting microbes. Another sugar fraction would become available to fermenting microorganisms only after digestion with hydrolytic enzymes (Fig. 1), which might be produced by the microorganisms themselves or exogenously added. Finally, the sum of these two fractions is further on referred to as “total bioaccessible sugars” (Fig. 1), representing both sugars that would immediately be available and those becoming available to fermenting microbes over time.

### Total reducing sugar determination based on the DNSA method

Amounts of total reducing sugars in aqueous extracts of solid substrates were determined using the dinitrosalicylic acid (DNSA) method (Bailey 1988; Gonçalves et al. 2010). Briefly, 25 µL DNSA reagent (1% (w/v) DNSA, 30% (w/v) potassium sodium tartrate, 1,6% (w/v) NaOH) was added to 25-µL sample in the wells of a 96-well microplate (flat bottom). Subsequently, the microplate (covered with a lid) was placed on the orbital shaker at 150 rpm for 30 s, followed by incubation in the oven at 85 °C for 10 min. After that, the microplate was cooled down on ice, and 250 µL of distilled water was immediately added to the wells. The absorbance was read at 530 nm, using a GENios Plus microplate reader (Tecan, Männedorf, Switzerland). Calibration of the method was carried out using a mixture of equal amounts of D-glucose and D-xylose, based on essentially comparable amounts of glucan and xylan in wheat straw as reported before (García-Torreiro et al. 2016).

### Determination of total sugars after acidic hydrolysis based on the phenol–sulphuric acid method

Amounts of total sugars in aqueous extracts of solid substrates were photometrically determined after acidic hydrolysis as previously described (Singh et al. 2014; Duong et al. 2022a), based on the phenol–sulphuric acid method (Dubois et al. 1956).

### Ultra-performance liquid chromatography (UPLC) analysis of individual sugars in lignocellulosic samples

Aqueous supernatants (0.5 mL) of samples arising from the enzymatic digestion and second extraction step (Fig. 1) were placed in 1.5-mL Eppendorf tubes, supplemented with 0.5 mL acetonitrile, thoroughly mixed, and stored at − 20 °C until further use. Before analysis, samples were centrifuged at 20,817 × g and 4 °C for 10 min (Eppendorf centrifuge 5430-R). Aliquots (3.3 μL) of the resulting supernatants were directly subjected to an Aquity™ UPLC system (Waters, Eschborn, Germany) comprising of a binary solvent manager (BSM), a sample manager (SM), and an evaporative light scattering detector (ELSD) and equipped with an Acquity UPLC BEH Amide column (1.7 μm particle size; 2.1 × 100 mm; Waters) operated at a column temperature of 35.0 °C. The ELSD conditions were gain, 500; data rate, 10 pps; gas pressure, 30.0 psi; mode, cooling; and drift tube temperature, 50.0 °C. The following solvents served as mobile phases: solvent A, 80/20 acetonitrile/water and 0.2% TEA (triethylamine), and solvent B, 50/50 acetonitrile/water and 0.2% TEA. The following elution profile was applied: isocratic elution at 1% B for 0.18 min, linear increase to 99% B until 10.00 min, isocratic elution at 99% B until 10.25 min, linear decrease to 1% B until 10.50 min, and isocratic elution at 1% B until 13.00 min (0.130 mL/min flow rate).

Calibration of the method was carried out using external standards. Glucose, xylose, and fructose showed the best matches with retention times of peaks in samples and were applied for calibration in a concentration range of 16 to 2000 mg/L. Other sugars such as arabinose, galactose, mannose, maltose, sorbitol, and mannitol were also tested but did not yield sufficient matches with peak retention times in samples. Representative UPLC-ELSD chromatograms of glucose, xylose, and fructose in both a standard mixture and a sample, related calibration curves derived from exponential regression, and an overview of the retention times of different sugars in UPLC analyses can be found in the Supplementary Information (Figs. S1, S2, S3, and Table S1). Calibrations based on external standards were included in each analysis run, also to ensure the identity of detected peaks as these displayed slight shifts in retention times in different analytical runs (Supplementary Fig. S1 and S2, and Table S1).

### Isothermal microcalorimetry

The isothermal microcalorimetry approach underlying the present work was previously comprehensively described (Duong et al. 2022a,b). Briefly, heat production rates of fungal wheat straw cultures in calorimetric vials were determined against reference vials containing 3 mL of sterile tap water, using the MC CAL isothermal microcalorimeter mentioned before at 28.000 ± 0.001 °C. The aforementioned amount of water was chosen to ensure that the heat capacity of the sample and the reference were approximately equal. In order to ensure a sufficient oxygen concentration and to prevent the accumulation of produced CO_2_ in the calorimetric vials, the latter were aseptically opened from a quarter-minute to a half-minute on each day of the working week (i.e. from Monday to Friday). The software OriginPro 2020 (OriginLab Corp., Northampton, MA, USA) was used to evaluate the calorimetric signals (Duong et al. 2022a,b). Metabolic heat- (i.e. the integral of the heat production rate of fungal wheat straw cultures over time) related parameters such as metabolic heat yield coefficients (*Y*_*Q*/*X*_) derived from Duong et al. (2022a,b) were used to investigate possible correlations with amounts of sugars in the investigated water-extractable sugar fractions.

### Statistical analyses

Linear and non-linear correlation analyses were performed using OriginPro 2020 as outlined in the text. Unpaired two-sample (two-sided) Student’s *t*-tests were performed using Microsoft® Excel® 2013 (version 15.0.5327.1000; Microsoft Corporation, Redmond/WA, USA).

## Results

### Water-soluble sugars in differently treated lignocellulose fractions

**Fig. 2:**
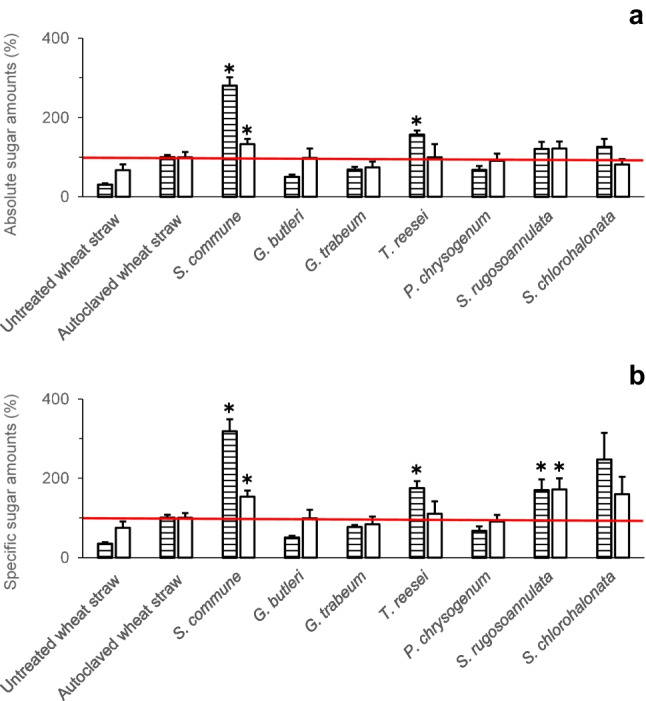
Percentages of absolute (mg) (**a**) and dry mass–specific (mg/g total dry mass at the end of the respective cultivation period) (**b**) sugar amounts (%) of untreated (not autoclaved) and autoclaved wheat straw without fungal pretreatment, respectively, and of previously autoclaved wheat straw after fungal pretreatment. Readily bioavailable sugars (horizontally striped bars) and total bioaccessible sugars (white bars) were determined using the phenol–sulphuric acid method (please refer to the section on experimental procedures for the definition of “readily bioavailable sugars” and “total bioaccessible sugars”). Sugar amounts (%) are expressed relative to amounts of corresponding sugars derived from autoclaved wheat straw (the horizontal red line denotes the 100% control value, respectively). Symbols and error bars represent means and standard deviations (calculated according to Gaussian error propagation rules) for triplicate cultures, respectively. Asterisks denote significant differences between fungal treatment and the autoclaved wheat straw control, respectively, according to unpaired two-sample (two-sided) Student’s *t*-tests (*P* < 0.05; *n* = 3). The sugar amounts in terms of absolute (mg) and specific quantities (mg/g), which served as the basis for the calculations of the percentage sugar amounts shown in this figure, can be found in the Supplementary Information (Tables S2 and S3)

The effects of fungal pretreatment of wheat straw on amounts of total sugars (potentially representing mono-, di-, oligo-, or polysaccharide fractions or combinations thereof) in differently generated aqueous wheat straw extracts (Fig. 1) were analysed. Both untreated (without autoclaving and fungal pretreatment) and autoclaved wheat straws (without fungal pretreatment) were used for comparison. Figure 2 depicts the percentages of absolute and specific (i.e. total dry mass–related) sugar amounts of untreated and of autoclaved wheat straw without fungal pretreatment, respectively, as well as of previously autoclaved wheat straw after fungal pretreatment. Readily bioavailable and total bioaccessible sugars (please refer to Fig. 1 for an explanation) were determined in aqueous extracts using the phenol–sulphuric acid method, which employs acidic hydrolysis of di-, oligo-, and polysaccharides potentially being present (please also refer to the corresponding sub-section of the experimental procedures). Sugar percentages are expressed relative to amounts of corresponding sugars derived from untreated and autoclaved wheat straw, respectively. The underlying raw sugar data for the calculations of the percentage sugar amounts can be found in the Supplementary Information (Tables S2 and S3).

Overall, amounts of readily bioavailable sugars were found to be considerably increased above the values of untreated wheat straw already by autoclaving, hereby additionally contributing to increased sugar amounts observed after pretreatment with certain fungal strains as can be inferred from the corresponding horizontally striped bars in Fig. 2a and b, respectively. A further significant increase in the readily bioavailable sugar fraction exceeding the effect of autoclaving was observed after fungal pretreatment with *S. commune* and *T. reesei* in terms of absolute and *S. commune*, *T. reesei*, and *S. rugosoannulata* in terms of specific amounts (please refer to the horizontally striped bars in Fig. 2a and b, respectively). Considering the amounts of total bioaccessible sugars, fungal pretreatment with *S. commune* and *S. rugosoannulata* followed by enzymatic digestion resulted in an increase in the absolute amounts of the total bioaccessible sugars by about 33% and 22%, respectively, compared to control values derived from autoclaved wheat straw without fungal pretreatment (white bars in Fig. 2a). The related quantities of total bioaccessible sugars amounted to about 139 and 127 mg for *S. commune* and *S. rugosoannulata*, respectively (corresponding to approximately 287 and 321 mg/g, respectively, with respect to the total dry mass–related sugars), while figures for untreated and autoclaved wheat straw of around 70 and 104 mg, respectively, were found (corresponding to about 140 and 187 mg/g with regard to the total dry mass–related sugars; Supplementary Tables S2 and S3; the related percentages of the dry mass–specific amounts of the total bioaccessible sugars are depicted in Fig. 2b).

**Fig. 3:**
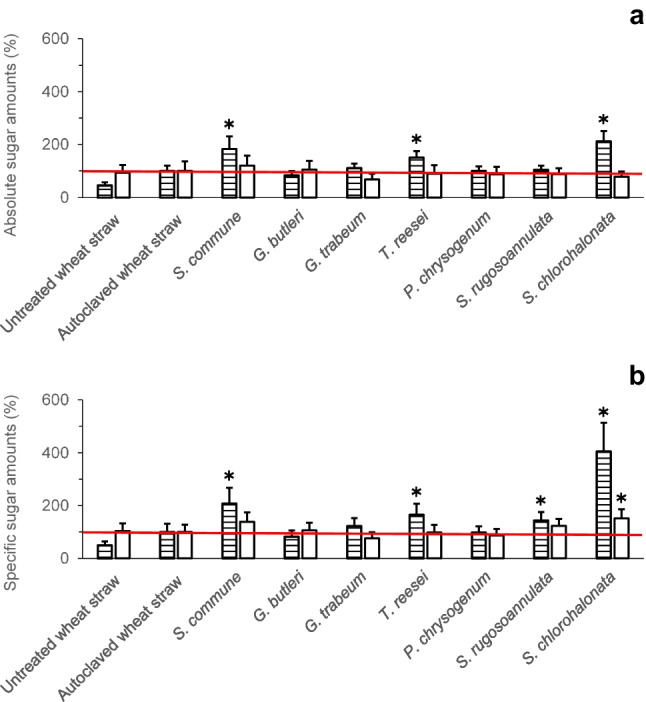
Percentages of absolute (mg) (**a**) and dry mass–specific (mg/g total dry mass at the end of the respective cultivation period) (**b**) sugar amounts (%) of untreated (not autoclaved) and autoclaved wheat straw without fungal pretreatment, respectively, and of previously autoclaved wheat straw after fungal pretreatment. Readily bioavailable sugars (horizontally striped bars) and total bioaccessible sugars (white bars) were determined using the DNSA method (please refer to the section on experimental procedures for the definition of “readily bioavailable sugars” and “total bioaccessible sugars”). Sugar amounts (%) are expressed relative to amounts of corresponding sugars derived from autoclaved wheat straw (the horizontal red line denotes the 100% control value, respectively). Symbols and error bars represent means and standard deviations (calculated according to Gaussian error propagation rules) for triplicate cultures, respectively. Asterisks denote significant differences between fungal treatment and the autoclaved wheat straw control, respectively, according to unpaired two-sample (two-sided) Student’s *t*-tests (*P* < 0.05; *n* = 3). The sugar amounts in terms of absolute (mg) and specific quantities (mg/g), which served as the basis for the calculations of the percentage sugar amounts shown in this figure, can be found in the Supplementary Information (Tables S4 and S5)

The percentages of absolute and specific (i.e. total dry mass–related) sugar amounts as determined with the DNSA method, which detects reducing ends of mono-, di-, oligo-, or polysaccharides potentially being present, are displayed in Fig. 3. The underlying raw sugar data for the calculations of the percentage sugar amounts can be found in the Supplementary Information (Tables S4 and S5). Fungal pretreatment with *S. commune* followed by enzymatic digestion always resulted in higher amounts of sugars (about 20% and 39% in terms of the absolute and specific total bioaccessible sugar amounts, respectively), as compared to control values derived from autoclaved wheat straw without fungal pretreatment (please refer to the corresponding white bars in Fig. 3a and b). Similar to the results obtained from sugar analysis employing the phenol–sulphuric acid method (Supplementary Tables S2 and S3), treatments followed by enzymatic digestion always yielded higher quantities of the thereof derived total bioaccessible sugars as compared to the corresponding amounts of readily bioavailable sugars where the final enzymatic digestion step was omitted (Supplementary Tables S4 and S5). As expected, the amounts of sugars determined with the DNSA method (Supplementary Tables S4 and S5) were always lower than the corresponding values determined with the phenol–sulphuric acid method (Supplementary Tables S2 and S3) since the phenol–sulphuric acid method detects the sum concentration of sugars after acidic hydrolysis, hereby capturing also sugar monomers released from di-, oligo-, or polysaccharides (Dubois et al. 1956). In contrast, the DNSA method only determines the concentration of free reducing ends of sugars and does not detect monomeric sugar units bound in oligo- or polysaccharide chains (Bailey 1988; Gonçalves et al. 2010).

### Effects of wheat straw pretreatment on enzymatic digestibility

**Fig. 4:**
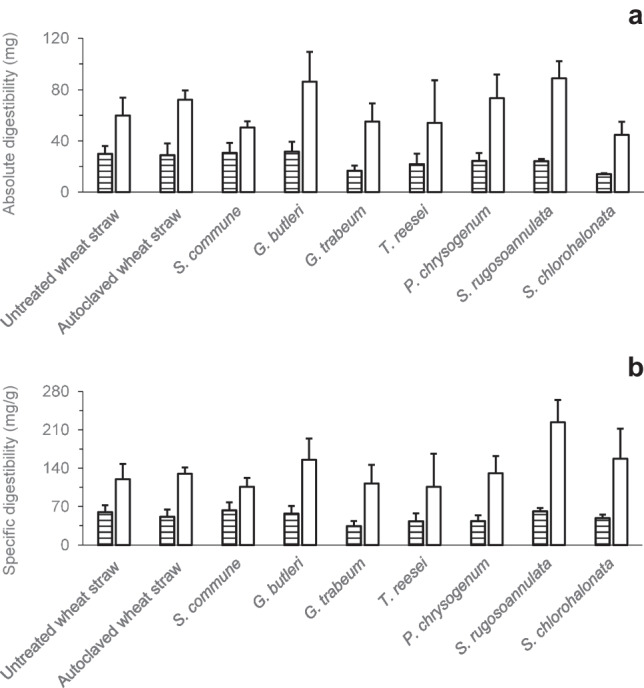
Absolute (mg) (**a**) and specific digestibility (mg/g total dry mass at the end of the respective cultivation period) (**b**) of untreated (not autoclaved) and autoclaved wheat straw without fungal pretreatment, respectively, and of previously autoclaved wheat straw after fungal pretreatment. Sugars were determined using the DNSA (horizontally striped bars) and phenol–sulfuric acid method (white bars), respectively. Symbols and error bars represent means and standard deviations (calculated according to Gaussian error propagation rules) for triplicate cultures, respectively. The absolute (mg) and specific sugar amounts (mg/g) underlying the digestibility calculations can be found in the Supplementary Information (Tables S2–S5)

**Fig. 5:**
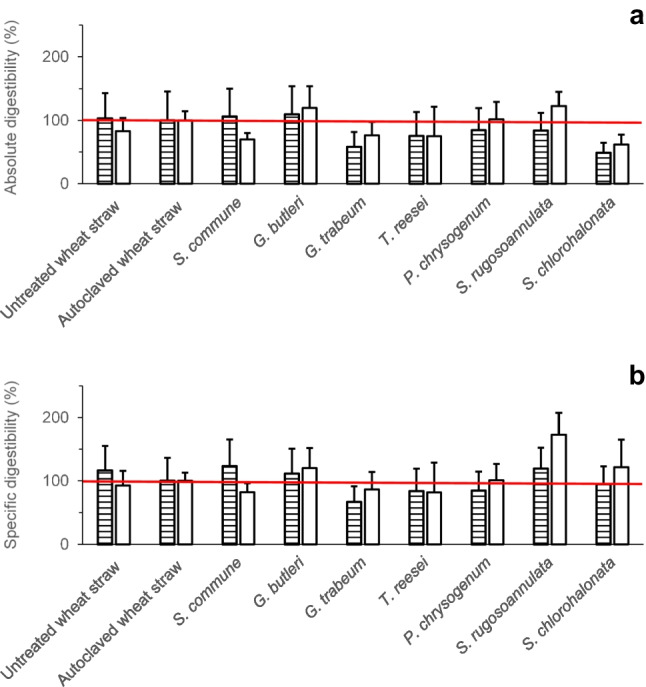
Percentages of absolute (mg) (**a**) and specific (mg/g total dry mass at the end of the respective cultivation period) (**b**) enzymatic digestibility (%) of untreated (not autoclaved) and autoclaved wheat straw without fungal pretreatment, respectively, and of previously autoclaved wheat straw after fungal pretreatment. Sugars were determined using the DNSA (horizontally striped bars) and phenol–sulphuric acid method (white bars), respectively. The percentages of the absolute and dry mass–specific digestibility were expressed relative to corresponding digestibility derived from autoclaved wheat straw (the horizontal red line denotes the 100% control value, respectively). Symbols and error bars represent means and standard deviations (calculated according to Gaussian error propagation rules) for triplicate cultures, respectively. The absolute (mg) and specific sugar amounts (mg/g) underlying the digestibility calculations can be found in the Supplementary Information (Tables S2–S5)

The effects of fungal pretreatment on the enzymatic digestibility of wheat straw (defined here as the accessibility of wheat straw for degradation by hydrolytic enzymes, and recorded as the increase in water-soluble sugar fractions after enzymatic digestion) were investigated. The digestibility of untreated wheat straw and of autoclaved wheat straw without fungal pretreatment was analysed for comparison. The enzymatic digestibility was calculated based on the sugar amounts derived upon application of the phenol–sulphuric acid method and also based on the corresponding values obtained from employment of the DNSA method (Figs. 4 and 5). The absolute digestibility (Fig. 4a) was calculated on the basis of absolute amounts of sugars as the difference between sugar amounts in aqueous extracts with and without enzymatic digestion, respectively (Fig. 1), while for the specific digestibility, this difference was expressed on the basis of the corresponding specific (i.e. total dry mass–related) sugar amounts.

Application of the phenol–sulphuric acid method yielded absolute enzymatic digestibility of about 60 and 72 mg for untreated and autoclaved wheat straw, respectively (Fig. 4a), thereby demonstrating an improved digestibility due to autoclaving. The highest absolute digestibility was reached after pretreatment with *S. rugosoannulata* (approximately 89 mg), followed by *G. butleri* (about 86 mg). Pretreatment with the other fungi did not lead to increased digestibility, compared to that of autoclaved wheat straw (Fig. 4a). The values of absolute enzymatic digestibility as determined with the DNSA method were always lower than those based on application of the phenol–sulphuric acid method (Fig. 4a). Comparable digestibility of about 30 and 29 mg was observed for untreated and autoclaved wheat straw, respectively. The highest DNSA measurement–based absolute digestibility was obtained after pretreatment with *G. butleri* (approximately 32 mg; Fig. 4a).

Specific (i.e. total dry mass–related) enzymatic digestibility based on the phenol–sulphuric acid method amounted to approximately 120 and 130 mg/g for untreated and autoclaved wheat straw, respectively (Fig. 4b). The highest specific digestibility was reached after pretreatment with *S. rugosoannulata* (about 224 mg/g), followed by *S. chlorohalonata* (around 157 mg/g), and *G. butleri* (approximately 155 mg/g). Similar to the absolute enzymatic digestibilities (Fig. 4a), the values of the specific digestibilities derived from application of the DNSA method were always lower than those obtained with the phenol–sulphuric acid method. The highest DNSA measurement–based specific digestibility amounted to about 63 mg/g (observed with *S. commune*; Fig. 4b) whereas corresponding figures for untreated and autoclaved wheat straw of about 60 and 51 mg/g, respectively, were recorded (Fig. 4b).

A comparison of the percentages of the absolute (Fig. 5a) and specific enzymatic digestibility (Fig. 5b) revealed that *S. rugosoannulata* and *G. butleri* yielded the highest absolute digestibility values as determined with the phenol–sulphuric acid method (about 123% and 120%, respectively, of the control value of autoclaved wheat straw without fungal pretreatment; Fig. 5a). With regard to the specific digestibility, control-exceeding values of approximately 173% and 120% were recorded for the aforementioned fungi, respectively (Fig. 5b). As observed for the digestibility in terms of absolute (Fig. 4a) and specific values (Fig. 4b), also the percentage digestibility that was based on the phenol–sulphuric acid method was higher than that derived from the DNSA method (Fig. 5).

The enzymatic digestibility of sugars present in the form of polymers (further on shortly referred to as “polymeric sugar digestibility”) was calculated as the difference between the enzymatic digestibility derived from application of the phenol–sulphuric acid method and the corresponding value obtained with the DNSA method, respectively (Table 1). All values of Table 1 exceeding 100% indicate a successful fungal pretreatment in terms of an improved digestibility of polymeric sugars going beyond the digestibility-improving effect of autoclaving. With respect to practical applications, especially the absolute polymeric sugar digestibility is important because it shows what could really be derived from comparable starting amounts of wheat straw. The highest absolute polymer digestibility (calculated using the corresponding absolute amounts of sugars) was observed with *S. rugosoannulata*, followed by *G. butleri* and *P. chrysogenum* (Table 1).

**Table 1:** Absolute (mg) and specific enzymatic digestibility (mg/g total dry mass at the end of the respective cultivation period) of polymeric sugars of untreated (not autoclaved) and autoclaved wheat straw without fungal pretreatment, respectively, and of previously autoclaved wheat straw after fungal pretreatment. For calculation of the digestibility of polymeric sugars, the enzymatic digestibility of a sample determined upon application of the DNSA method was subtracted from that derived from application of the phenol–sulphuric acid method, respectively. The percentages of the absolute and specific polymer digestibility, which were expressed relative to the corresponding digestibility derived from autoclaved wheat straw, are also shown. Data always refer to means ± standard deviations (calculated according to Gaussian error propagation rules, respectively) for triplicate cultures. The absolute (mg) and specific sugar amounts (mg/g) underlying the digestibility calculations can be found in the Supplementary Information (Tables S2–S5)

	Absolute polymeric sugar digestibility				Specific polymeric sugar digestibility			
		(mg)		(%)	(mg/g)			(%)
	Mean	SD	Mean	SD	Mean	SD	Mean	SD
Untreated wheat straw	30.2	15.2	69.5	40.8	60.4	71.0	77.0	111.1
Autoclaved wheat straw	43.4	13.2	100.0	43.0	78.4	65.5	100.0	118.2
*S. commune*	18.7	10.6	43.2	27.6	41.5	92.2	52.9	125.6
*G. butleri*	54.7	24.0	126.1	67.3	98.3	76.7	125.4	143.4
*G. trabeum*	38.4	14.4	88.4	42.7	77.7	60.4	99.2	113.2
*T. reesei*	32.4	34.0	74.6	81.6	62.9	85.9	80.3	128.5
*P. chrysogenum*	49.1	17.9	113.2	53.7	87.3	62.3	111.4	122.4
*S. rugosoannulata*	64.4	15.3	148.4	57.2	162.7	91.9	207.6	209.4
*S. chlorohalonata*	30.7	13.9	70.7	38.5	108.4	136.7	138.3	209.2

Aqueous supernatants of samples arising from enzymatic digestion (Fig. 1) were further analysed for their individual sugar composition by UPLC coupled to ELSD. Peaks of presumable sugars in UPLC-ELSD chromatograms of wheat straw samples (Supplementary Fig. S2) were compared with those observed with UPLC-ELSD analysis of external sugar standards (Supplementary Fig. S1, Supplementary Table S1). The peaks derived from glucose, xylose, and fructose standards yielded the best matches in retention time patterns with presumable sugar peaks recorded in wheat straw samples, respectively, and were used for sugar identification and quantification in the samples (Supplementary Fig. S3). Other tested sugars (arabinose, galactose, mannose, maltose, sorbitol, mannitol; Supplementary Table S1) displayed only unsatisfactory retention time matches with presumable sugar peaks in samples and were therefore not further considered.

The results of sugar analyses in terms of absolute and specific amounts of glucose, fructose, and xylose for untreated and autoclaved wheat straw as well as after fungal pretreatment derived from aqueous extraction of the straw in combination with enzymatic digestion are depicted in Supplementary Fig. S4a and b, respectively. As expected, glucose was always the clearly dominating cellulose-related monosaccharide, followed by fructose and the hemicellulose-derived pentose xylose. The sum of all sugars detected by UPLC-ELSD analysis after enzymatic hydrolysis in combination with aqueous extraction (i.e. glucose, fructose and xylose; Supplementary Fig. S4a and S4b) was always higher than the corresponding sum values derived from application of the DNSA method (which detects free reducing ends of sugars; Supplementary Tables S4 and S5) at varying individual extents. The DNSA assay is known to respond differently to various types of sugars and is further affected by the degree of sugar polymerisation (Breuil and Saddler 1985; Shao and Lin 2018), which impedes a direct comparison of corresponding amounts of sugars derived from UPLC-ELSD and DNSA analysis, respectively.

### Correlation between metabolic heat yield coefficients and amounts of water-soluble sugars in differently treated lignocellulose fractions

Metabolic heat yield coefficients (*Y*_*Q*/*X*_) previously determined for growth of the seven investigated fungal strains on wheat straw (Duong et al. 2022a, b) were used to investigate possible correlations between these metabolic heat–related parameters and the amounts of water-soluble sugars in differently treated wheat straw fractions (Fig. 1). In this respect, the total bioaccessible sugar fraction deserves particular consideration as it represents those sugars which potentially could be utilised after fungal pretreatment in follow-up processes, either immediately (aqueous-soluble sugars of the readily bioavailable sugar fraction) or after subsequent digestion of the pretreated lignocellulosic material by polysaccharide-cleaving enzymes.

**Fig. 6:**
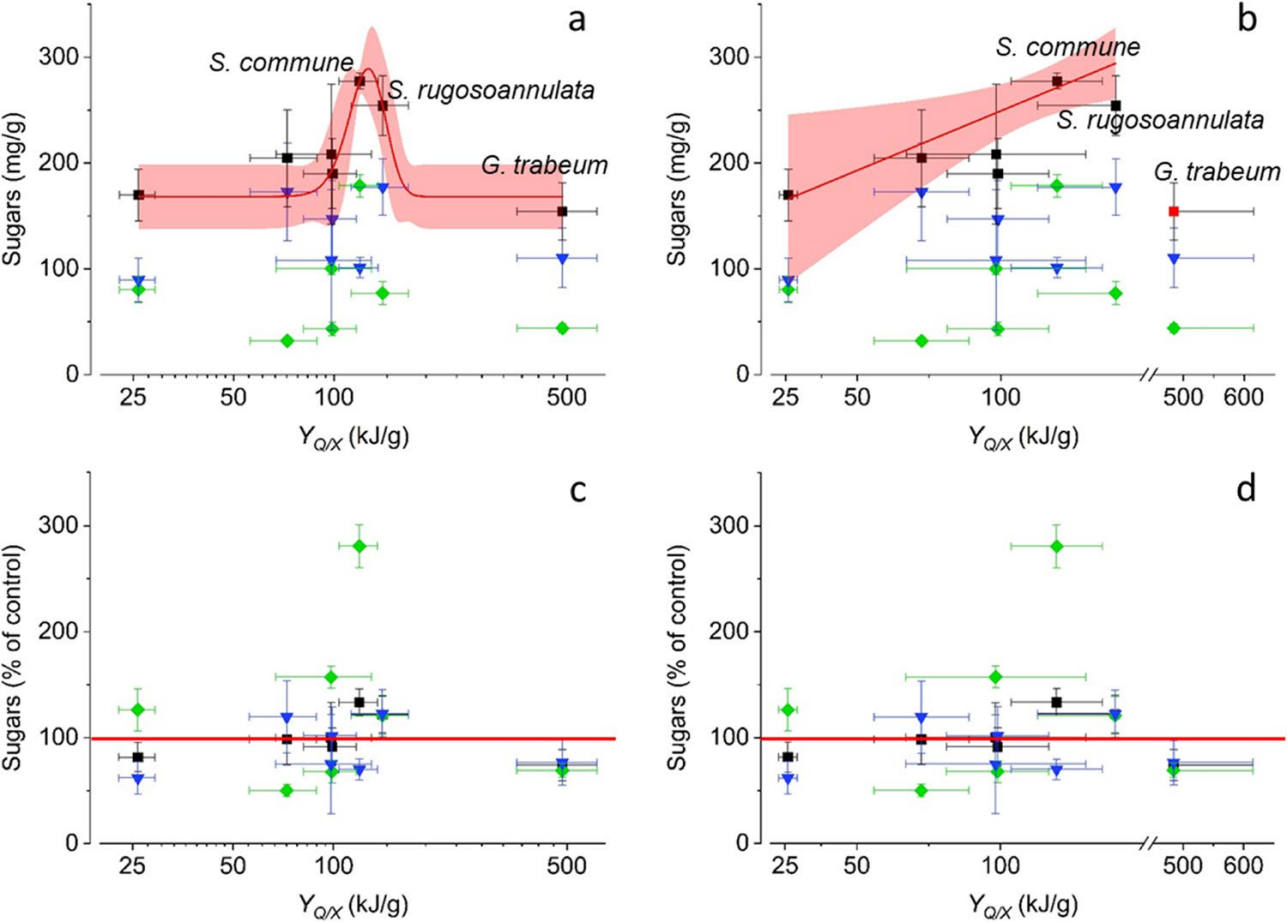
Total bioaccessible sugars (black squares), readily bioavailable sugars (green diamonds), and sugars derived from enzymatic digestion after fungal pretreatment (blue inverse triangles; the colour codes applied for these three sugar fractions correspond to those applied in Fig. 1) as function of the metabolic heat yield coefficient (*Y*_*Q*/*X*_) for wheat straw after pretreatment with the seven investigated fungi, respectively. Amounts of sugars were expressed based on a uniform dry mass of 0.5-g wheat straw initially applied (mg/g) (**a**, **b**), and relative to corresponding control values derived from autoclaved wheat straw without fungal pretreatment (% of control) (**c**, **d**; the horizontal red lines denote the 100% control values, respectively). Sugar amounts were determined using the phenol–sulphuric acid method. Logarithmic scaling of *x* axes (**a**–**d**) and *x* axis interruption (**b**, **d**) was chosen to facilitate reading, respectively. Symbols correspond to experimentally determined parameters derived from fungal wheat straw cultures and represent means ± standard deviations (calculated according to the Gaussian error propagation rules) for triplicate cultures. The sugar amounts serving as a basis for Fig. 6 can be found in the Supplementary Information (Tables S2 and S3), and the *Y*_*Q*/*X*_ values were taken from Duong et al. (2022a,b). The solid red lines in **a** and **b** result from non-linear fitting employing the GaussAmp model of OriginPro 2020 (coefficient of determination *R*^2^ > 0.97) and linear fitting of the experimentally determined data (*R*^2^ > 0.75; curve fit forced through zero), respectively (the data set of the brown-rot basidiomycete *G. trabeum* is indicated by a red square in **b** and was excluded from linear fitting due to the divergent Fenton chemistry–based cellulose degradation mechanism of this fungus). The 95% confidence bands corresponding to data fitting in **a** and **b** are labelled in pink, respectively. The names of the fungi where pretreatment led to an increase in total bioaccessible sugars above the control value derived from autoclaved wheat straw (*S. commune* and *S. rugosoannulata*) and also that of *G. trabeum* are highlighted in **a** and **b**

Figure 6 depicts the amounts of sugars of the differently treated wheat straw fractions as functions of the corresponding *Y*_*Q*/*X*_ values of the seven investigated fungi, respectively. In order to ensure comparability with respect to process efficiency, amounts of sugars were expressed based on a uniform dry mass of 0.5-g wheat straw initially applied (mg/g) (Fig. 6a, b) and also relative to corresponding control values derived from autoclaved wheat straw without fungal pretreatment (% of control) (Fig. 6c, d; the horizontal red lines indicate the 100% control values, respectively). Sugar amounts were determined using the phenol–sulphuric acid method, and logarithmic scaling and *x* axis interruption were applied to facilitate reading in Fig. 6a and c and in Fig. 6b and d, respectively. The sugar amounts serving as a basis for Fig. 6 can be found in the Supplementary Information (Tables S2 and S3). An *Y*_*Q*/*X*_ range between about 120 and 140 kJ/g was suggested to delimit optimal conditions for pretreatment (i.e. those yielding maximal amounts of total bioaccessible sugars) upon non-linear Gaussian fitting (Fig. 6a). Among the tested fungi, *S. commune* and *S. rugosoannulata* fall into this range and both yielded total bioaccessible sugar amounts exceeding the 100% control value (Fig. 6a and c). A further data analysis was conducted, where the data of *G. trabeum* were excluded and linear regression was applied (Fig. 6b). The rationale for excluding *G. trabeum* from data fitting is that this brown-rot basidiomycete applies a lignocellulose decomposition mechanism being different from those of all other fungi. Brown-rot fungi such as *G. trabeum* do not employ complete cellulase systems for accessing the polymeric sugars of lignocellulose and use a biological Fenton-type reaction instead (Riley et al. 2014; Krueger et al. 2017; Bissaro et al. 2018). A fairly good linear correlation (*R*^2^ > 0.75) between *Y*_*Q*/*X*_ and the amounts of total bioaccessible sugars was obtained for the remaining fungi (Fig. 6b; the corresponding relative values are shown in Fig. 6d), which are known to use common cellulase systems and not polysaccharide cleavage via extracellular Fenton chemistry to access polymeric sugars (Riley et al. 2014; Duong et al. 2022b, 2022a). In this context, it must be noted that the models applied for data fitting have no underlying conceptual value. They have simply been employed as quantitative descriptors of the potential relationships between the investigated parameters.

## Discussion

In the present study, we aimed at the applicability of calorimetry for the process monitoring of fungal SSF-based pretreatment of lignocellulosic agricultural by-products. To this end, wheat straw was employed as a proxy for a globally important feedstock along with a range of filamentous fungi representing different lignocellulose decay types.

In order to characterise the efficiency of wheat straw pretreatments in SSF processes with respect to the accessibility of sugars for potential subsequent use, water-extractable sugar fractions possibly representing mono-, di-, oligo-, or polysaccharides or combinations thereof were first analysed in differently treated lignocellulose fractions derived from pretreatment/SSF experiments (Fig. 1). Both water-soluble sugars being immediately available after fungal pretreatment and those becoming water-extractable after enzymatic digestion of the pretreated wheat straw with hydrolysing (hemi)cellulases were considered to constitute the total bioaccessible sugar fraction, which was taken as a measure indicating the success of the pretreatments. In line with previous findings (Collins et al. 2014; Tishler et al. 2015), glucose, fructose, and xylose were always the most prominent sugar monomers found in aqueous extracts of differently treated wheat straw (Supplementary Figs. S2 and S4).

The observation that the lignin-degrading white-rot fungi *S. rugosoannulata* and *S. commune*—the latter representing a basidiomycete intermediate between brown-rot and white-rot fungi (Riley et al. 2014)—led to the most efficient fungal pretreatments exceeding the corresponding control values in terms of both absolute and specific amounts of total bioaccessible sugars (Fig. 2a, b; Supplementary Tables S2 and S3) corroborates previous findings. It has long been indicated that delignification is an important prerequisite for the efficient hydrolytic saccharification of lignocellulosic feedstock and hence represents a major challenge with respect to the successful utilisation of lignocellulosic wastes (Singh et al. 2014; Andlar et al. 2018). In this regard, white-rot fungi are the primary lignin degraders in nature and their lignin degradation capabilities clearly surpass those of brown-rot and soft-rot fungi (Andlar et al. 2018; Weng et al. 2021). White-rot basidiomycetes are therefore attractive biocatalysts for pretreatment of lignocellulosic biomasses and hold promise for high sugar yields upon enzymatic saccharification. For instance, successful application of the white-rot fungus *Trametes hirsuta* for pretreatment of paddy straw and corn stover was described before (Sun et al. 2011; Saritha et al. 2012). The white-rot fungus *I. lacteus* was employed for the biological pretreatment of cornstalk, which was accompanied by the production of various extracellular hydrolytic and oxidative enzymes (Du et al. 2011).

Considering the economic efficiency of a pretreatment process, the absolute amounts of total bioaccessible sugars remaining after pretreatment are decisive for the potential further utilisation in follow-up processes. In this respect, the brown-rot basidiomycete *G. trabeum* was not effective in pretreating wheat straw during an incubation time of 20 days (Fig. 2a). *G. trabeum* is perhaps the best understood brown rot fungus (Cohen et al. 2005), including its genome that has been sequenced (Floudas et al. 2012). Contradicting effects of *G. trabeum* were previously reported for pretreatment of corn fibre (Shrestha et al. 2009), wood chips (Monrroy et al. 2010), aspen (Schilling et al. 2012), bamboo (Xu et al. 2013), and wheat straw (Hermosilla et al. 2018). During saccharification of spruce or pine previously degraded for up to 4 weeks by *G. trabeum*, a maximum actual cellulose-to-glucose conversion efficiency of only 10.6% was reached (Schilling et al. 2009). In addition to the respective lignocellulosic substrate used, also the incubation time used for pretreatment with this fungus was found to have a large influence. Whereas a comparatively short pretreatment time of 10 days resulted in an increased sugar yield compared to the control, longer incubation times significantly decreased the sugar recovery (Hermosilla et al. 2018). Our observation that also the tested soft-rot ascomycetes and the mucoromycete *G. butleri* did not increase the absolute amounts of the total bioaccessible sugars above the control value (Fig. 2a) is in line with previous results, where no enhancing effects of pretreatments using a soft-rot ascomycete (*Trichoderma viride*) and a mucoromycete (*Mucor* sp.) on enzymatic saccharification of softwood biomass could be observed (Ray et al. 2010).

Individual patterns regarding (i) amounts of sugars determined in the differently treated lignocellulosic fractions with the phenol–sulfuric acid vs. the DNSA method (Figs. 2, 3, 4, and 5), (ii) effects of the pretreatments on enzymatic digestibility (Figs. 4 and 5, and Table 1), and (iii) absolute vs. dry mass–specific sugar amounts and enzymatic digestibility (Figs. 2, 3, 4, and 5, Table 1) observed upon wheat straw pretreatment with the investigated fungi indicate individual fungal characteristics with respect to the respective ecological strategy employed during lignocellulose utilisation and the underlying enzyme inventory and growth behaviour. These fungal characteristics have already comprehensively been described before (Duong et al. 2022a,b). The ratio of sugar amounts determined with the phenol–sulfuric acid vs. the DNSA method (the latter only responding to free reducing ends of sugars; Bailey 1988; Gonçalves et al. 2010) should reflect the extent of cleavage of polysaccharide chains at the time point of analysis, with increasing numbers of cleavage sites leading to increased amounts of sugars becoming accessible to determination with the DNSA method. For instance, for the autoclaved wheat straw control, the amount of readily bioavailable sugars determined with the DNSA method accounted for only about 19% of the value of the same sugar fraction when analysed with the phenol–sulfuric acid method (Supplementary Tables S2 and S4). The share of the DNSA method-based amount of the readily bioavailable sugar fraction was only increased to approximately 23% upon pretreatment with the comparatively slow-growing basidiomycete *S. rugosoannulata*, while being much more extensively increased to about 39% after pretreatment with the fast-growing ascomycetous mould *S. chlorohalonata* (Supplementary Tables S2 and S4; for a more detailed description of the characteristics of these fungi, please refer to Duong et al. (2022a,b)). Increases in specific (i.e. total dry mass–based) amounts of the total bioaccessible sugars above the corresponding 100% control values as also observed for pretreatment with *S. chlorohalonata* in addition to *S. commune* and *S. rugosoannulata* (Fig. 2b, 3b; Supplementary Tables S3 and S5) indicate an improvement of the quality of the lignocellulosic substrate with respect to subsequent accessibility and thus usability of remaining sugars. However, for *S. chlorohalonata*, such potential benefits were clearly counteracted by a comparatively rapid sugar consumption along with rapid growth of this fungus (Duong et al. 2022a,b). These growth and substrate consumption characteristics obviously impede a successful use of *S. chlorohalonata* for pretreatment as suggested by the remaining amounts of total bioaccessible sugars for subsequent use, which did not exceed the 100% control values (Figs. 2a and 3a). Similar limitations exist with respect to further partial improvements of the substrate quality resulting from pretreatment with various fungi, where increases in enzymatic digestibilities above the corresponding control values were observed (Figs. 4 and 5; Table 1). Also in such cases, the amounts of total bioaccessible sugars finally remaining for further use did not exceed control values detected for autoclaved wheat straw (Figs. 2a and 3a). The observed effects of wheat straw autoclaving on both absolute and specific amounts of readily bioavailable sugars (Figs. 2 and 3) and absolute and specific enzymatic digestibility of polymeric sugars (Table 1) resemble effects known from steam explosion pretreatment of lignocellulosic biomass. During this mechanic-physico-chemical pretreatment process, lignocellulose is first exposed to saturated steam at high pressure and temperature, followed by a sudden pressure drop, which finally results in disruption of the lignocellulose fibrous structure and a strong increase of cellulose enzymatic digestibility (Ziegler-Devin et al. 2021).

Together with previously published data (Salvachúa et al. 2011; Tuyen et al. 2012; Singh et al. 2014), the aforementioned findings indicate that delignification is not the only important parameter to be considered for the assessment of the suitability of different fungal species for pretreatment of lignocellulosic substrates. Examples for further functional attributes, which can considerably influence the efficiency and feasibility of fungal pretreatment, include (i) fungal growth rates, (ii) the extent and spectrum of lignocellulosic sugar consumption for fungal growth and energy conservation, and in this context also fungal effects on the digestibility of cellulose and hemicellulose remaining after pretreatment; (iii) the potential production of inhibitory compounds, or (iv) fungal combativeness and antagonistic abilities against potential competitors (Salvachúa et al. 2011; Boddy and Hiscox 2016; Duong et al. 2022b). Fungal activity parameters based on metabolic heat measurements accompanying fungal growth on wheat straw such as metabolic heat yield coefficients (*Y*_*Q*/*X*_) are indicative of the degree of investment of the lignocellulosic resource into fungal biomass production, which is at the expense of investment into other functional attributes contributing to lignocellulose decomposition such as those mentioned before (Duong et al. 2022b). With respect to activity parameters reflecting the extent of resource channelling into biomass production, we found *Y*_*Q*/*X*_ values to be much more robust and less error-prone than corresponding biomass yield coefficients (*Y*_*X*/*S*_) in a previous study (Duong et al. 2022b). We therefore used fungal *Y*_*Q*/*X*_ values in order to investigate possible correlations between these metabolic heat–related parameters and the amounts of water-soluble sugars in differently treated wheat straw fractions. Whereas an *Y*_*Q*/*X*_ range between approximately 120 and 140 kJ/g was suggested to be optimal for pretreatment upon consideration of all investigated fungi and application of a non-linear Gaussian fitting model (Fig. 6a), a linear relationship where amounts of total bioaccessible sugars were suggested to increase with increasing *Y*_*Q*/*X*_ values was obtained upon exclusion of the brown-rot basidiomycete *G. trabeum* from analysis (Fig. 6b). Different from all other investigated fungi, *G. trabeum* uses a biological Fenton-type lignocellulose decomposition mechanism for accessing the polymeric sugars of lignocellulose, instead of employing complete cellulase systems as other fungi do (Riley et al. 2014; Krueger et al. 2017; Bissaro et al. 2018). It remains to be elucidated in further in-depth investigations employing a broader range of fungi whether a *Y*_*Q*/*X*_ range being optimal for fungal pretreatment could firmly be established, maybe irrespective of the fungal lignocellulose decay mechanism employed; or if the sugar accessibility for post-treatment uses generally increases with increasing *Y*_*Q*/*X*_ values as long as “conventional” enzymatic, i.e. (hemi)cellulase-based, lignocellulose decomposition mechanisms are active.

Our results suggest that metabolic heat measurement–derived parameters such as *Y*_*Q*/*X*_ values may become very valuable tools supporting the assessment of the suitability of different fungal species for pretreatment of lignocellulosic substrates. Metabolic heat measurements may also be applied to record fungal growth stages during lignocellulose pretreatment processes, based on a linear correlation between fungal biomass formation and the corresponding metabolic heat released (Duong et al. 2022a,b). Further studies should also address the suitability of metabolic heat measurements and fungal activity parameters derived thereof for the optimisation of incubation times to be employed for pretreatment and the applicability of biocalorimetric approaches with respect to monitoring of the progress of pretreatment processes that are conducted under non-sterile conditions.

## Supplementary Information

Below is the link to the electronic supplementary material.

Supplementary file1 (PDF 279 KB)

## Data Availability

The data supporting the findings of this study are available from the corresponding author on request.
