# Supplementary material for: Biocalorimetry-aided monitoring of fungal pretreatment of lignocellulosic agricultural residues
Source: Appl Microbiol Biotechnol. 2024 Jun 25;108:394. doi: 10.1007/s00253-024-13234-y (PMC11199272; doi:10.1007/s00253-024-13234-y)
Supplement: Supplementary file 1 [file 253_2024_13234_MOESM1_ESM.pdf]

Applied Microbiology and Biotechnology

Supplementary Information

Biocalorimetry-aided monitoring of fungal pretreatment of lignocellulosic agricultural residues

Hieu Linh Duong [a, b], Sven Paufler [a], Hauke Harms [a], Thomas Maskow [a, *], Dietmar Schlosser [a, *]

[a] Department of Applied Environmental Microbiology, Helmholtz-Centre for Environmental Research - UFZ, Permoserstraβe 15, 04318 Leipzig, Germany

[b] Vietnamese-German University (VGU), Ring Road 4, Quarter 4, Thoi Hoa Ward, Ben Cat City, Binh Duong Province, Vietnam

* Correspondence: dietmar.schlosser@ufz.de (D.S.)

Content

Fig. S1 2
Fig. S2 3
Fig. S3 4
Fig. S4 5
Table S1 6
Table S2 7
Table S3 8
Table S4 9
Table S5 10

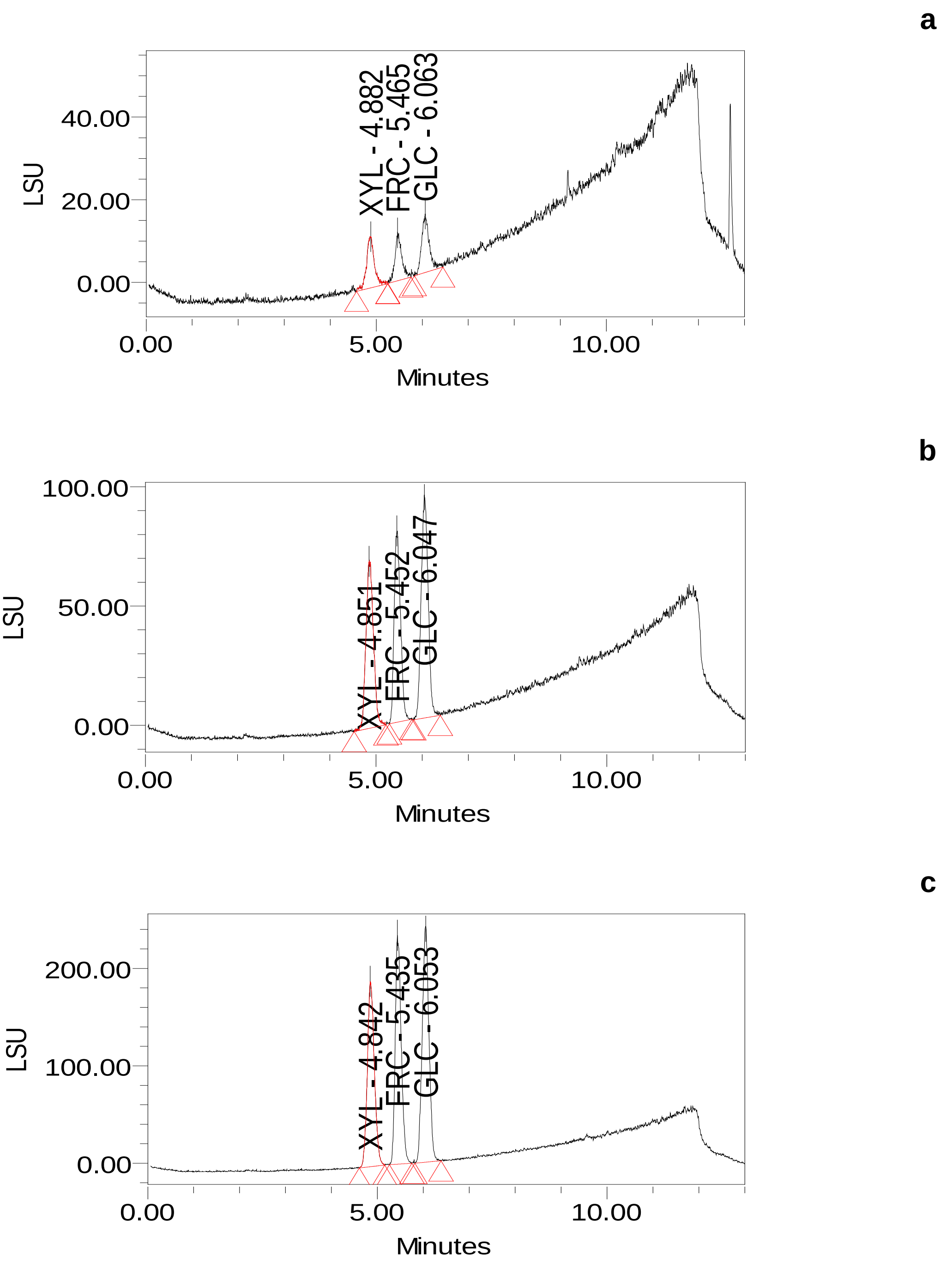

Fig. S1 Representative UPLC-ELSD chromatograms of 3 sugar standards applied in mixture at (a) 62.5 mg/L, (b) 250 mg/L, and (c) 500 mg/L, respectively. Peak labels (associated with the corresponding retention times): XYL, xylose; FRC, fructose; GLC, glucose.

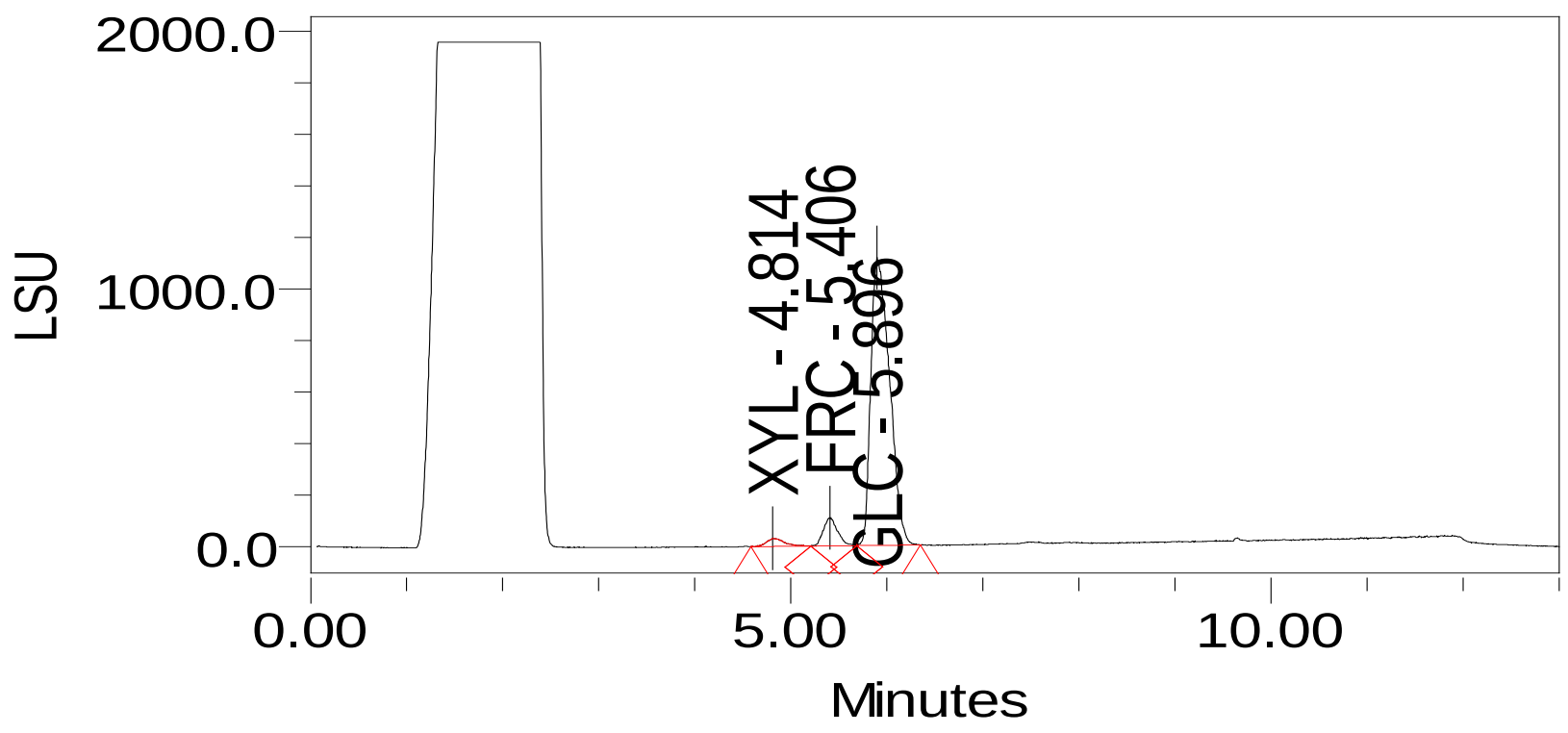

Fig. S2 Representative UPLC-ELSD chromatogram of a sample derived from autoclaved wheat straw after enzymatic digestion. The detected sugars are indicated by the peak labels XYL for xylose, FRC for fructose, and GLC for glucose. The corresponding retention times are also shown. External sugar standards were included in each analysis run in order to ensure the identity of the detected sugar peaks, which displayed slight shifts in retention times in different analytical runs (please also refer to Fig. S1 and Table S1 for comparison).

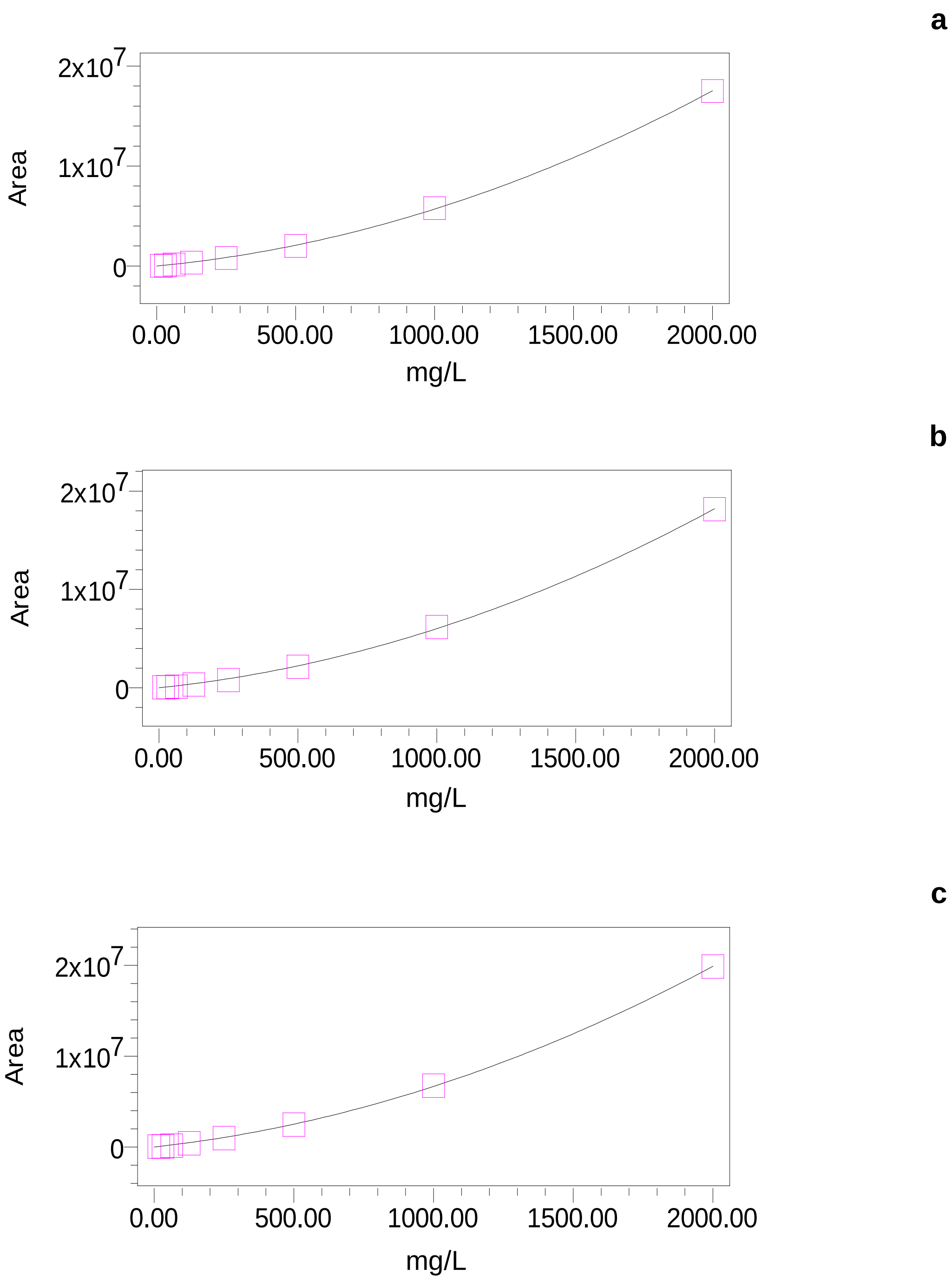

Fig. S3 Representative exponential calibration curves for (a) xylose, (b) fructose, and (c) glucose derived from UPLC-ELSD analysis of sugar standard mixtures, as exemplified in Fig. S1. The corresponding coefficients of determination (R^2) were always > 0.99.

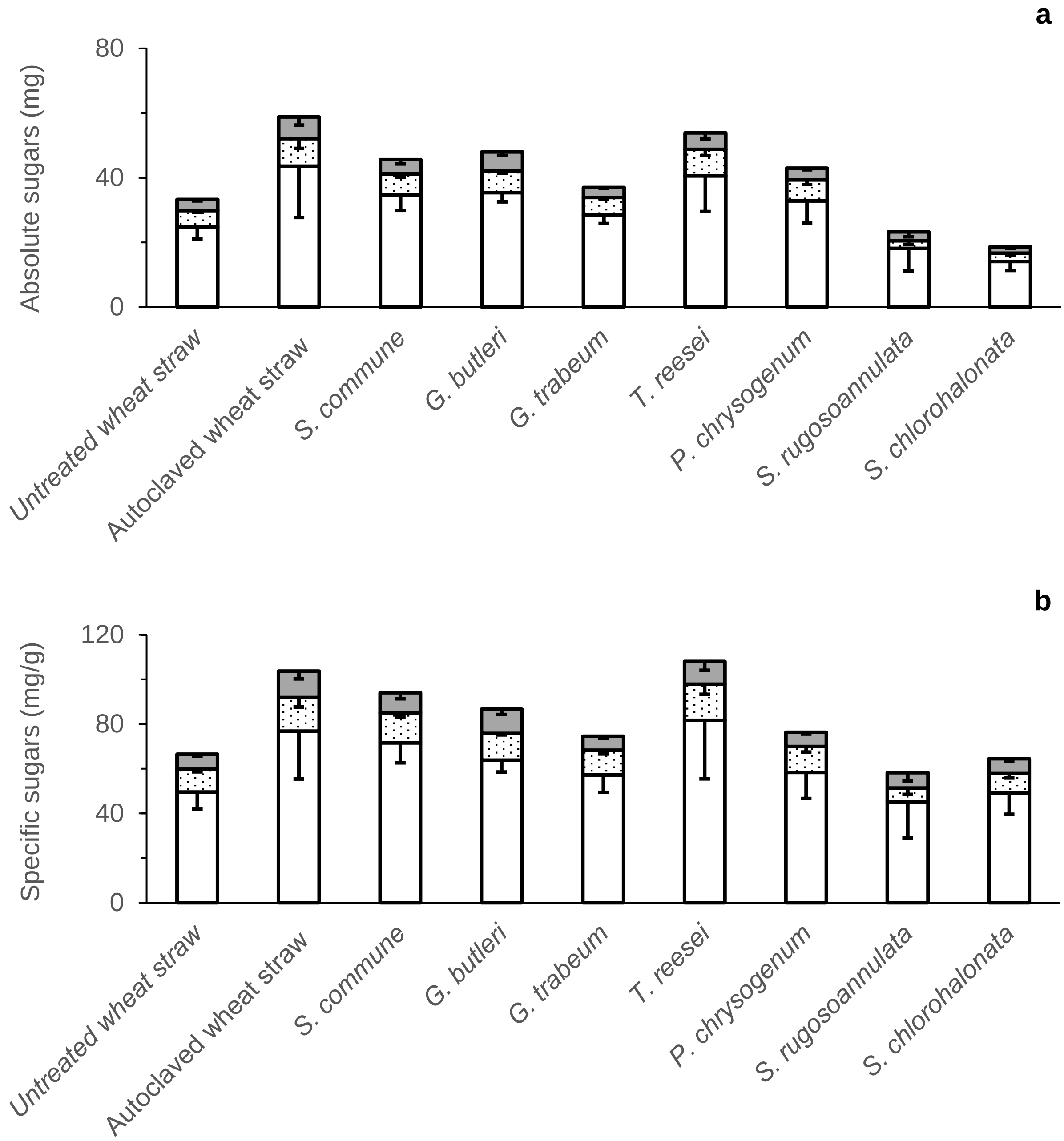

Fig. S4 Absolute (mg) (a) and specific (i.e. total dry mass-related; mg/g) (b) amounts of glucose (white bars), fructose (dotted bars), and xylose (grey bars) determined using UPLC-ELSD analysis for untreated (not autoclaved) and autoclaved wheat straw without fungal pretreatment, and for wheat straw after fungal pretreatment. Wheat straw samples were enzymatically digested prior to analysis, and aqueous extracts derived thereof (please refer to Fig. 1 of the materials and methods section) were analysed. Symbols and error bars represent means and standard deviations for triplicate cultures, respectively.

Table S1 Representative retention times of various sugar standards subjected to UPLC-ELSD analysis.

Compound	Retention time (min) [1]
Glucose [2]	5.903
Fructose [2]	5.489
Xylose [2]	4.877
Cellobiose	7.753
Maltose	7.764
Sorbitol	5.988
Arabinose	5.066
Mannose	5.764
Galactose	6.189

(1) Representative retention times derived from singe UPLC-ELSD runs are shown, respectively.

(2) The peaks of glucose, xylose and fructose standards showed the best retention time matches with presumable sugar peaks observed in real samples, respectively, and were therefore used for sugar identification and quantification in the samples. Other tested sugars (arabinose, galactose, mannose, maltose, sorbitol, mannitol) displaying only unsatisfactory retention time matches with presumably sugar peaks in samples were not further considered.

Table S2 Absolute sugars derived from the first extraction with McIlvaine buffer, the second extraction of solids with Na-citrate buffer only, and the second extraction of solids with Na-citrate buffer accompanied by enzymatic digestion (please refer to Fig. 1 of the materials and methods section). Amounts of sugars were determined using the phenol-sulphuric acid method. Data refer to means and standard deviations (SD) for triplicate cultures, respectively.

	First extraction with McIlvaine buffer (mg)		Second extraction with Na-citrate buffer only (mg)		Second extraction with enzymatic digestion (mg)	
	Mean	SD	Mean	SD	Mean	SD
Untreated wheat straw	NA[1]		9.8	0.9	69.7	13.8
Autoclaved wheat straw	28.5	2.4	3.3	1.6	75.5	7.2
S. commune	39.0	2.9	49.2	2.5	99.7	4.1
G. butleri	12.6	1.2	3.3	0.6	89.7	23.1
G. trabeum	12.4	1.6	9.5	1.1	64.7	14.1
T. reesei	37.8	0.2	12.3	2.6	66.4	33.1
P. chrysogenum	17.2	1.8	4.3	1.4	77.8	18.3
S. rugosoannulata	20.9	2.0	17.5	5.3	106.1	12.3
S. chlorohalonata	32.2	3.4	8.0	3.9	52.7	9.5

[1] NA, not applicable.

Table S3 Dry mass-specific amounts of total sugars (mg/g total dry mass at the end of the respective cultivation period) derived from the first extraction with McIlvaine buffer, the second extraction of solids with Na-citrate buffer only, and the second extraction of solids with Na-citrate buffer accompanied by enzymatic digestion (please refer to Fig. 1 of the materials and methods section). Amounts of sugars were determined using the phenol-sulphuric acid method. Data refer to means and standard deviations (SD) for triplicate cultures, respectively.

	First extraction with McIlvaine buffer (mg/g)		Second extraction with Na-citrate buffer only (mg/g)		Second extraction with enzymatic digestion (mg/g)	
	Mean	SD	Mean	SD	Mean	SD
Untreated wheat straw	NA[1]		19.6	1.8	139.5	27.6
Autoclaved wheat straw	51.2	4.5	5.9	2.9	135.5	11.2
S. commune	80.5	5.8	100.2	7.1	206.1	14.3
G. butleri	22.7	1.3	6.0	0.8	161.2	38.6
G. trabeum	24.8	2.2	19.1	1.8	130.8	34.2
T. reesei	75.3	4.1	24.5	5.7	130.2	60.2
P. chrysogenum	30.7	3.4	7.7	2.5	138.2	31.2
S. rugosoannulata	52.8	6.5	44.0	13.1	267.8	38.3
S. chlorohalonata	113.0	27.2	28.2	14.6	185.3	52.7

[1] NA, not applicable.

Table S4 Absolute sugars derived from the first extraction with McIlvaine buffer, the second extraction of solids with Na-citrate buffer only, and the second extraction of solids with Na-citrate buffer accompanied by enzymatic digestion (please refer to Fig. 1 of the materials and methods section). Amounts of sugars were determined using the DNSA method. Data refer to means and standard deviations (SD) for triplicate cultures, respectively.

	First extraction with McIlvaine buffer (mg)		Second extraction with Na-citrate buffer only (mg)		Second extraction with enzymatic digestion (mg)	
	Mean	SD	Mean	SD	Mean	SD
Untreated wheat straw	NA[1]		2.8	0.6	32.6	6.3
Autoclaved wheat straw	5.6	0.9	0.6	0.4	29.4	9.3
S. commune	7.8	1.4	3.6	2.6	34.2	7.4
G. butleri	4.3	0.8	0.9	0.1	32.5	7.7
G. trabeum	5.6	0.7	1.3	0.3	18.1	3.9
T. reesei	8.0	0.2	1.4	0.5	23.2	8.3
P. chrysogenum	5.0	0.3	1.2	0.2	25.5	6.3
S. rugosoannulata	4.8	0.3	1.7	0.2	25.9	1.8
S. chlorohalonata	12.5	1.8	0.7	0.2	14.8	0.6

[1] NA, not applicable.

Table S5 Dry mass-specific amounts of total sugars (mg/g total dry mass at the end of the respective cultivation period) derived from the first extraction with McIlvaine buffer, the second extraction of solids with Na-citrate buffer only, and the second extraction of solids with Na-citrate buffer accompanied by enzymatic digestion (please refer to Fig. 1 of the materials and methods section). Amounts of sugars were determined using the DNSA method. Data refer to means and standard deviations (SD) for triplicate cultures, respectively.

	First extraction with McIlvaine buffer (mg/g)		Second extraction with Na-citrate buffer only (mg/g)		Second extraction with enzymatic digestion (mg/g)	
	Mean	SD	Mean	SD	Mean	SD
Untreated wheat straw	NA[1]		5.6	1.2	65.1	12.5
Autoclaved wheat straw	10.2	2.5	1.1	0.7	52.3	13.1
S. commune	16.2	2.9	7.4	5.2	70.4	13.3
G. butleri	7.7	1.7	1.6	0.0	58.6	13.9
G. trabeum	11.3	2.0	2.6	0.5	36.5	9.4
T. reesei	15.9	1.2	2.9	1.1	45.7	14.5
P. chrysogenum	9.0	0.5	2.2	0.4	45.3	10.8
S. rugosoannulata	12.0	0.5	4.3	0.3	65.3	6.3
S. chlorohalonata	43.4	7.1	2.6	0.8	51.3	6.3

[1] NA, not applicable.
